# Hybrid Ultrathin Gold Nanowire Gels: Formation and Mechanical Properties

**DOI:** 10.1002/smll.202411506

**Published:** 2025-03-03

**Authors:** Yannic Curto, Srishti Arora, Bart‐Jan Niebuur, Lola González‐García, Tobias Kraus

**Affiliations:** ^1^ INM – Leibniz Institute for New Materials Campus D2 2 66123 Saarbrücken Germany; ^2^ Department of Materials Science and Engineering Saarland University 66123 Saarbrücken Germany; ^3^ Colloid and Interface Chemistry Saarland University 66123 Saarbrücken Germany

**Keywords:** colloidal gel, rheology, SAXS, ultrathin gold nanowires

## Abstract

This report is about the chemical formation of gels from ultrathin gold nanowires (AuNWs) and the gels’ properties. An excess of triphenylphosphine (PPh_3_) initiated the gelation of AuNWs with core diameters below 2 nm and an oleylamine (OAm) ligand shell dispersed in cyclohexane. The ligand exchange of OAm by PPh_3_ changes the AuNW‐solvent interactions and leads to phase separation of the solvent to form a macroscopic gel. Small angle X‐ray scattering and transmission electron microscopy indicate that hexagonal bundles in the original dispersion are dispersed, and the released nanowires entangle. Rheological analyses indicate that the resulting gel is stabilized both by physical entanglement and crosslinking of AuNWs by Van der Waals and π–π interactions. Chemically formed AuNW gels have solid‐like properties and crosslinks that distinguish them from highly concentrated non‐crosslinked AuNW dispersions. The AuNW gel properties can be tuned via the Au:PPh_3_ ratio, where smaller ratios led to stiffer gels with higher storage moduli.

## Graphical abstract

The formation of colloidal gels of ultrathin gold nanowires is chemically induced due to a ligand exchange after the addition of triphenylphosphine. During the gelation process, the bundle arrangement of the nanowires decomposes, and the wires entangle. Rheological analysis shows that besides entanglement, also crosslinks stabilize the gel structure, and gel stiffness can be tuned with the amount of triphenylphosphine.

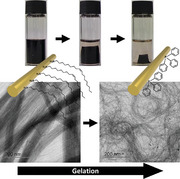

## 1 Introduction

Ultrathin gold nanowires (AuNWs) are 1D nanostructures with diameters of only a few nanometers and aspect ratios on the order of 10^3^. Their metallic, electrically conductive core makes them suitable for nanoelectronics, sensors, and catalysis. For example, Gong et al. created a wearable pressure sensor based on AuNW‐coated tissue paper.^[^ ^1^ ^]^ Maurer and coworkers printed AuNWs with elastomer stamps to fabricate transparent, flexible electronics.^[^ ^2^, ^3^ ^]^ Theoretical calculations and experimental results show that AuNWs can used for sensing of pyridine, CH_3_SH, CH_3_NH_2_, CH_3_CHO, CO, and other molecules due to a resistance change when they adsorb on the wire surface.^[^ ^4^, ^5^, ^6^ ^]^ Zhu et al. showed that AuNWs are suitable as electro‐catalysts for the reduction of CO and CO_2_.^[^ ^7^ ^]^

Ultrathin AuNWs can be synthesized by the reduction of chloroauric acid in the presence of oleylamine (OAm).^[^ ^8^, ^9^, ^10^ ^]^ The resulting ultrathin wires have gold cores with diameters of 1.6 to 1.9 nm (6 to 7 gold atoms) surrounded by an organic ligand shell of OAm with a thickness similar to the core diameter.^[^ ^8^, ^9^, ^10^, ^11^ ^]^ They tend to self‐assemble into superstructures called “bundles”.^[^ ^12^ ^]^ These colinear wire assemblies were first observed in the originally published synthesis of the AuNWs^[^ ^10^ ^]^ in *n*‐hexane and then studied in more detail and for other solvents.^[^ ^12^, ^13^ ^]^ Loubat et al. first identified hexagonally ordered AuNW bundles in the liquid state via in situ Small Angle X‐ray Scattering (SAXS) during synthesis.^[^ ^13^ ^]^ Gao et al. showed that AuNW bundle formation in linear alkanes is entropically driven,^[^ ^12^ ^]^ Bettscheider et al. elucidated the role of unbound ligands in the entropic AuNW assembly.^[^ ^11^ ^]^ The exact geometry of the bundles depends on AuNW concentration, solvent type, ligand type, ligand density, and the concentration of free ligand molecules.^[^ ^11^, ^12^, ^13^, ^14^, ^15^, ^16^ ^]^ All regular AuNW bundles that have been reported exhibited hexagonal packing, with average center‐to‐center‐spacings in n‐hexane of *d*_c─c_ ≈ 9.2 nm directly after synthesis. Purification by washing in n‐hexane reduced *d*_c─c_ in hexane to ≈5.5 nm.^[^ ^15^ ^]^ Bundles in cyclohexane had *d*_c─c_ ≈ 5.9 nm; polar solvents reduced spacing, e.g. to *d*_c─c_ ≈ 4.1 nm for ethanol.^[^ ^12^ ^]^ Nouh and coworkers replaced OAm by trioctylphosphine (TOP), which reduced *d*_c─c_ in *n*‐hexane to 3.8 nm after 72h.^[^ ^14^ ^]^

Bundling can be used to create materials from AuNWs. Reiser and coworkers spun AuNWs into macroscopic fibers by injecting a nanowire dispersion into ethanol.^[^ ^17^ ^]^ The fibers had hierarchical superstructures and mechanical properties that depended on AuNW alignment. Shear flow in the nozzle increased alignment and led to fibers with a higher breaking stress.^[^ ^17^ ^]^ Lu et al. created twisted assemblies of AuNW bundles in rope‐like structures that they induced by Pd coating of the nanowires.^[^ ^18^ ^]^ Gong et al. produced stretchable supercapacitors with membranes made from AuNW bundles.^[^ ^19^ ^]^ Engel et al. showed that bundling affects the nanoimprint of electrodes from inks containing AuNW dispersions.^[^ ^20^, ^21^ ^]^ Chemically induced gelation has not yet been reported for AuNWs.

The gelation of metal nanospheres is well‐documented, and aerogels of spherical metal, metal oxide, and semiconductor nanoparticle gels are widely studied.^[^ ^22^, ^23^, ^24^ ^]^ Gelation can for example be induced by covalent crosslinking of the nanoparticles, by controlled destabilization of the dispersion, in a one‐pot self‐destabilization as in sol‐gel synthesis, or by subsequent destabilization of a stable colloidal dispersion via ionic strength, pH, surface polarity, solvent polarity, or solvent evaporation.^[^ ^22^ ^]^ For example, Bigall et al. synthesized noble metal particle gels (Au, Ag, AuAg) using hydrogen peroxide (H_2_O_2_) as a destabilizing reagent.^[^ ^25^ ^]^ Naskar et al. destabilized a Pt nanoparticle dispersion in *n*‐hexane with hydrazine that replaced the stabilizing surfactant OAm on the particle surface and induced gelation.^[^ ^26^ ^]^ The gelation of metal nanoparticles has been used for the production of metal aerogels that combine low density, electrical conductivity, high surface area, and high porosity.^[^ ^22^, ^27^ ^]^ Noble metal aerogels have been applied in electrocatalysis and optical sensing: Wen et al. demonstrated that Au aerogels functionalized with beta‐cyclodextrin could be used in electrocatalysis for glucose oxidation in the absence of enzymes.^[^ ^27^ ^]^ Gao et al. produced Au/Ag alloy aerogels for usage in surface enhanced Raman spectroscopy. They found significant enhancement of Raman signal intensities for Rhodamine 101 with the aerogel as compared to the precursor Au/Ag alloy nanoparticles.^[^ ^28^ ^]^

Gels of 1D nanostructures are less common, and many reports on organo‐ or hydrogels of ultrathin fibers focus on carbon nanotubes.^[^ ^29^, ^30^ ^]^ In 2012, Kong and coworkers published that Ag, Si, and MnO_2_ nanowires in ethanol assembled into 3D networks above certain concentrations due to Van der Waals forces at contact points of the nanowires and prepared electrically conductive aerogels from the gels by critical point drying.^[^ ^31^ ^]^ Subsequently, aerogels were prepared from nanowires made from Au, Ag, Cu, MnO_2_, TiO_2_, SiO_2_, SiC, and W_18_O_49_:^[^ ^23^, ^31^, ^32^, ^33^, ^34^, ^35^, ^36^ ^]^ Kong et al. used controlled solvent evaporation to prepare Ag and MnO_2_ nanowire gels.^[^ ^31^ ^]^ Cheng et al. used centrifugation to induce the gelation of ultrathin tungsten oxide nanowires.^[^ ^32^ ^]^ Qian et al. made aerogels from gold nanowires with core diameters of ≈7 nm by freeze‐casting nanowires in water – tert‐butanol mixtures.^[^ ^37^ ^]^ They obtained ultralow density Au aerogels and could tune their density, pore size, and geometry via solvent composition.^[^ ^37^ ^]^ Other reported methods of nanowire gelation include ice templating,^[^ ^38^ ^]^ crosslinker‐induced gelation,^[^ ^39^ ^]^ and in situ gelation, where synthesis and gelation of the nanowires are combined in one step.^[^ ^33^, ^40^ ^]^ These studies mainly focused on the production and characterization of aerogels; less is known about the properties of organo‐ or hydrogels of ultrathin 1D nanowires.

The gelation of ultrathin AuNWs (diameter below 2 nm) into a solid has not yet been reported. Saitoh et al. described highly viscos AuNW dispersions with 30 wt% Au content in *p‐tert*‐butyltoluene and reported non‐Newtonian liquid flow but no gelation.^[^ ^41^ ^]^ Nouh et al. reported that ligand exchange against phosphines (TOP) changed the bundling behavior but did not report on gelation.^[^ ^14^ ^]^ Qian et al. formed aerogels of thicker gold nanowires by directly freeze‐drying their AuNW suspensions but not report on hydro‐ or organogels.^[^ ^37^ ^]^

Here, we show that triphenylphosphine (PPh_3_) can replace OAm on the surface of ultrathin AuNW, change the surface polarity of the nanowires, and induce their gelation. We studied the network formation by reconstructing structural features of the changing nanowire network with Transmission Electron Microscopy (TEM) and in situ SAXS. The mechanical properties of the formed gel were characterized with rheology as a function of Au:PPh_3_ ratio. We report the tunable self‐assembly of gold nanowires into 3D network with gel‐like mechanical properties due to crosslinking and entanglement of the nanowires.

## 2 Results and Discussion

Ultrathin gold nanowires were synthesized according to a published protocol^[^ ^10^ ^]^ (see Experimental Section for details). They showed uniform core diameters of 1.6–1.9 nm according to SAXS analysis (Figure S1a, Supporting Information) and TEM images (Figure S1b,c, Supporting Information). Similar wires have been prepared previously; they typically had average lengths of several micrometers^[^ ^41^ ^]^ and OAm ligand shell densities of ≈4.5 ligands per nm^2^ after two washing steps.^[^ ^11^ ^]^ Purified AuNWs in cyclohexane formed hexagonal superstructures (bundles) with an average interwire distance of *d*_c─c_ ≈ 5.9 nm according to our SAXS results (Figure S1a, Supporting Information), in good agreement with previous reports.^[^ ^11^, ^12^ ^]^

**Figure 1:**
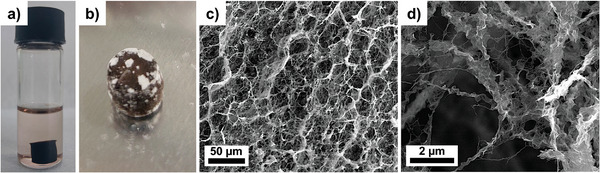
Formation and microstructure of a AuNW gel body in cyclohexane and freeze‐drying to form an aerogel. a) Gelation of a AuNW dispersion in cyclohexane with PPh_3_ (Au:PPh_3_ ≈ 1:100) after 18 h. b) The gels retained the shape of the vial and could be freeze‐dried to form an aerogel. c) Scanning electron micrograph of the overall and d) details of the porous wire network.

Gelation was initiated by mixing the AuNW dispersion with the same volume of a PPh_3_‐solution (266 mg mL^−1^) in cyclohexane in a sealed container. Visible phase separation of solvent and AuNW indicated the onset of gelation after 1 h for a Au:PPh_3_ ratio of 1:100 at room temperature. A solid gel was formed after ≈9 h. The resulting gel body had a dark brown color and retained the cylindrical shape of the vessel (**Figure** **1**a). It remained soft enough to deform when gently shaking it.

Several observations suggest that PPh_3_ replaces OAm on the AuNW surface and chemically induces gelation. First, we varied the PPh_3_ concentration and found that a molar ratio of Au:PPh_3_ < 1 was required for gelation to occur. Larger excess of PPh_3_ led to faster formation of a smaller volume of denser gel. Second, we found that all gels could be redispersed in methanol. The resulting dispersions contained agglomerates of intact AuNWs (TEM and optical micrographs in Figure S2, Supporting Information). This indicates a partially reversible, non‐covalent crosslinking mechanism that involves a change in polarity of the AuNW, consistent with ligand exchange. The non‐polar OAm is replaced by more polar PPh_3_.

Drying the gels in air led to collapse, shrinkage, and the formation of dense xerogels. Freeze‐drying of the gels preserved the overall shape and yielded porous aerogels with white inclusions of PPh_3_ crystals (Figure 1b) that precipitated during drying. Aerogels formed at Au:PPh_3_ ≈ 1:50 had a density of ≈44 kg m^−3^, larger than typical aerographites (2 kg m^−3^)^[^ ^42^ ^]^ and in the range of silica aerogels (3–350 kg m^−3^, often ≈ 100 kg m^−3^).^[^ ^43^ ^]^ The density of our aerogels was above that of aerogels made from thicker gold nanowires (6 – 23 kg m^−3^).^[^ ^37^ ^]^ This is presumably caused by the crystalline PPh_3_ impurities (white solid) visible in Figure 1b. The density of the pure aerogel fraction is almost certainly below our measured density. Scanning electron microscopy (SEM, Figure 1c,d) of the freeze‐dried aerogels showed a porous 3D network of aggregated nanowires. The network shown in Figure 1d shows the presence of nanoribbon‐ and nanofiber‐like structures. These nanostructures seem to be curved and partially entangled to form a porous network, similar to those described for aerogels of ultrathin W_18_O_49_ nanowires.^[^ ^32^ ^]^

We followed the gelation process by repeatedly removing samples until 240 min passed at Au:PPh_3_ ≈ 1:100. The samples were dried on a TEM grid, which caused collapse, and we imaged thin sections close to the edge of the dried network. Beam damage is known to fragment AuNW; we minimized it by reducing the duration of the electron beam irradiation. Note that while the resulting micrographs capture the typical nanoscale arrangement of the wires in bundles, the arrangement of the bundles are affected by the drying process, especially at the micrometer scale and above.

**Figure 2:**
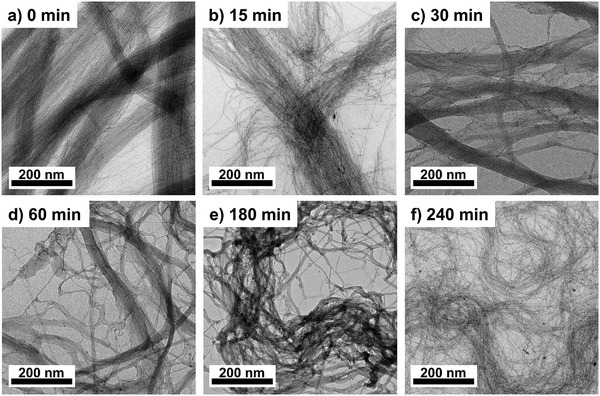
The gelation process of AuNWs is followed by TEM imaging. Elongated nanowire bundles rearranged into tangled nanowires while bundles dispersed. We followed this process by removing and drying samples at a) 0 min, b) 15 min, c) 30 min, d) 60 min, e) 180 min, and f) 240 min after PPh_3_ was added and gelation started.

The TEM micrographs in **Figure** **2**a show the AuNW bundles that were present directly after adding PPh_3_. The bundles shrank slowly in the first 60 min (Figure 2b–d); after 180 min (Figure 2e), most of the bundles contained only a few nanowires (Figure 2e). After 240 min, unbundled wires and entanglement of the nanowires were visible (Figure 2f). The average curvature of the AuNWs increased with time, suggesting the AuNW bundles became less stiff as bundle size decreased. This likely explains why thinner bundles curved around smaller radii and entangled after 180 min. It is remarkable that few wire fragments were visible even after 240 min. We conclude that gelation retains the wires’ structures. Bundles became thinner, sometimes down to single wires, and entangled. No further changes of the nanowires and their bundles could be seen in the TEM images after a gelation time of 1 day (Figure S3, Supporting Information).

**Figure 3:**
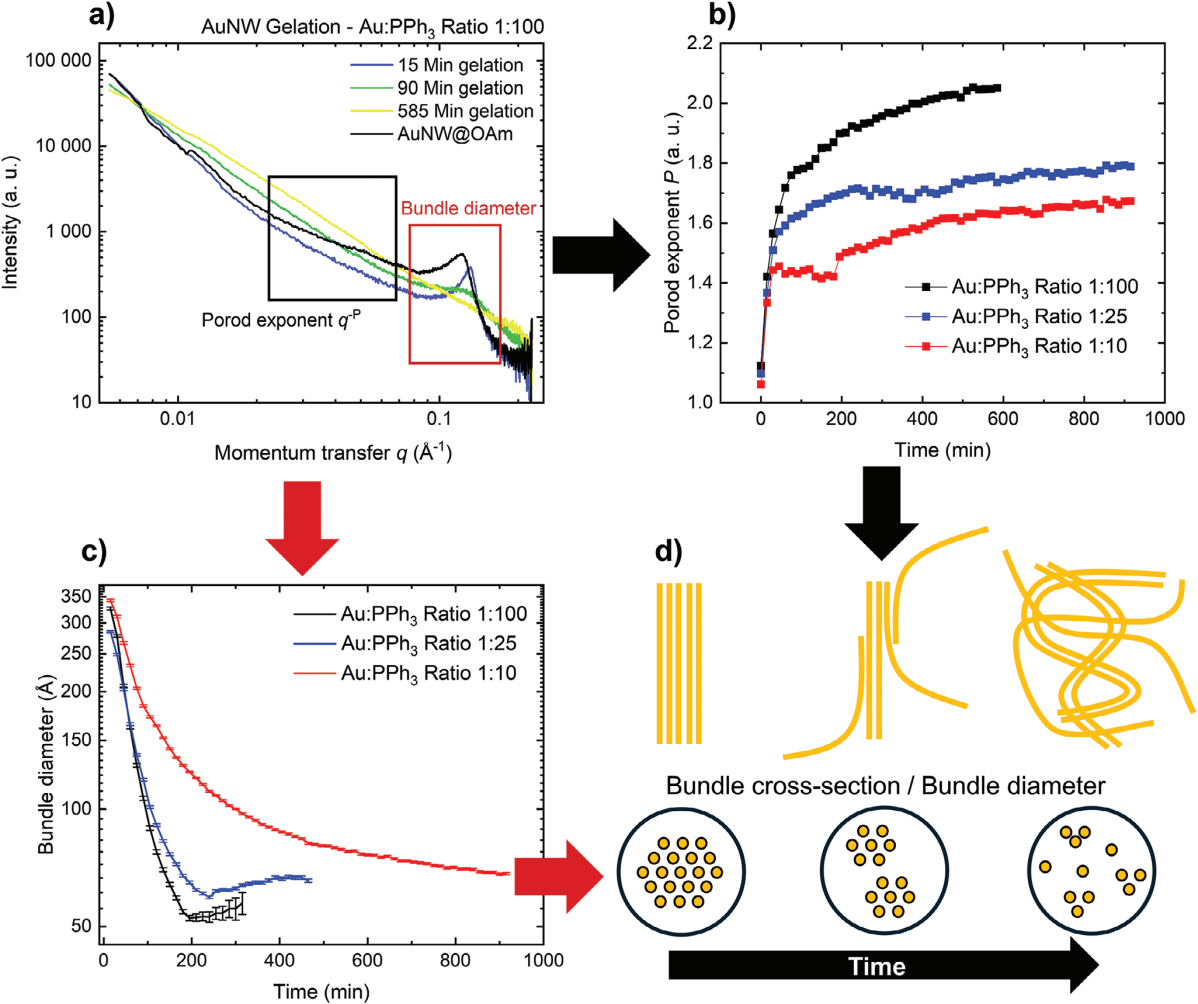
Bundle dispersion and entanglement of the AuNWs during the gelation as observed by in situ SAXS for different Au:PPh_3_ ratios. a) SAXS patterns obtained before (black trace) and during gelation with Au:PPh_3_ ≈ 1:100 at different gelation times. b) Time‐dependent Porod exponent (0.003–0.007 Å^−1^) during gelation at different Au:PPh_3_ ratios. c) Time‐dependent bundle diameter for different Au:PPh_3_ ratios. d) Proposed bundle decomposition and AuNW gelation mechanism.

We quantified the changes in wire and bundle structure during gelation at Au:PPh_3_ ≈ 1:100 by in situ SAXS (**Figure** **3**a). The slope of the scattering curve of pure AuNW@OAm dispersions in cyclohexane (black trace in Figure 3a) was modeled using a generalized Porod law given by Equation (1),

(1) $$I\left(q\right)∼q^{-P}$$

with *P* the Porod exponent. We found *P* = 1.09 in the Porod region between 0.03 and 0.07 Å^−1^, confirming the presence of nanowires with elongated cylindrical cores.^[^ ^44^ ^]^ A structure factor peak at 0.123 Å^−1^ (primary) indicated the presence of AuNW bundles with a wire spacing of *d*_c─c_  = 5.9 nm in accordance with literature.^[^ ^11^, ^12^, ^13^, ^15^ ^]^ Secondary and tertiary structure factor peaks at higher *q* values (Figure S4, Supporting Information) indicated a hexagonal superstructure of the bundles. These bundles had an average equivalent diameter of the cross‐section *D*_bundle_ of 30–40 nm.

The addition of PPh_3_ initiated changes of the average bundle diameter, the spacing of nanowires in the bundles, and the overall network structure of the bundles. The structure factor peak shifted from 0.123  to 0.131 Å^−1^ during 15 min. This indicates a reduction of wire spacing from *d*_c─c_  = 5.9  to 5.5 nm, which is readily explained by the exchange of OAm with PPh_3_. It is consistent with the observations by Nouh et al., who prepared TOP‐stabilized AuNW dispersions in *n*‐hexane and observed a shift from 5.5  to 4.85 nm after 30 min.^[^ ^14^ ^]^ They verified the exchange of OAm by TOP via NMR spectroscopy and DFT calculations and showed that the adsorption energy of phosphines to gold is larger than for amines.^[^ ^14^ ^]^ It is likely that PPh_3_ is able to replace OAm, too. The shifted wire spacing thus indicates a ligand exchange of OAm by PPh_3_ on the surface of the wires that started immediately after adding PPh_3_.

The structure factor peak at 0.131 Å^−1^ broadened and weakened during gelation and disappeared after ≈240 min. The structure factor peak was fitted with a model based on a form factor of a thin cylinder and structure factor for hexagonal arrangement (Equation S1, Supporting Information) that is described in detail in the Supporting Information. This revealed a decrease in *D*_bundle_ from ≈30–40 nm to ≈5–6 nm (Figure 3c), consistent with TEM observations of bundles with only a few nanowires. The final gel contained small, interconnected bundles of only a few wires and unbundled nanowires seen in the TEM images in Figure 2. Both are significantly more flexible and enable larger bending curvatures, as also can be seen in the TEM images.

The increase of the Porod exponent from the initial *P* = 1.09 to 1.37 after 15 min, 1.64 after 60 min, and 2.00 toward the end of the gelation process indicates a change of the mass fractal dimension of the nanowire network (Figure 3b). We interpret it in analogy to SAXS studies of polymer chains,^[^ ^45^ ^]^ where the change in slope is interpreted as a transition from elongated (swollen) to more tangled (compact) polymer chains. The nanowires switch from elongated, bundled arrangements to more tangled structures during gelation, consistent with the thinner, strongly curved bundles and wires visible in TEM images (Figure 2). Replacing OAm by PPh_3_ reduces nanowire‐solvent interactions, reduces bundle thickness and stiffness, enables a more tangled arrangement, phase separation, and cause shrinkage by solvent expulsion.

Note that alternative explanations of the increasing slope in the Porod would imply changes of the form factor. For example, the fragmentation of nanowires into spheres would cause changes in the slope, too.^[^ ^44^ ^]^ Such fragmentation can be caused by the Rayleigh‐Plateau‐instability that affects AuNWs.^[^ ^14^, ^46^, ^47^ ^]^ We cannot entirely exclude that such fragmentation occurs to a small extent. However, the TEM in Figure 2 suggests that most wires stay intact, and, thus, the change in slope is mainly caused by the network formation that underlies AuNW gelation.

Increasing the ratio to Au:PPh_3_ ≈ 1:25 and 1:10 in the gel formation led to similar SAXS curves (Figure S5, Supporting Information), but gel formation and bundle dissolution slowed down (Figure 3b,c). The rate of bundle diameter decrease ${\dot{D}}_{\mathrm{bundle}}$ dropped with increasing Au:PPh_3_ ratio (Figure 3c), suggesting that PPh_3_ concentration limited the rate of molecular ligand exchange. The difference of ${\dot{D}}_{\mathrm{bundle}}$ was larger between 1:10 and 1:25 than between 1:25 and 1:100, suggesting a limiting PPh_3_ concentration above which bundle decomposition does not speed up. This deviation from the standard law of mass action suggests that the ligand replacement mechanism follows a Langmuir or other adsorption isotherm with rapid initial PPh_3_ adsorption. Adsorption rates drop as OAm is increasingly replaced by PPh_3_ and steric hinderance limit adsorption sites. In addition, it is conceivable that bundles are already dispersed when only a fraction of OAm is replaced on their constituent wires, and that higher PPh_3_ excess leads to the exchange of a larger ligand fraction that speeds up bundle dissolution.

The final gel structures and properties changed with Au:PPh_3_, too. The structure factor peak at *q * =  0.131 Å^−1^ did not fully disappear at the end of gelation for Au:PPh_3_ ≈ 1:10 (Figure S5a, Supporting Information), and the macroscopic sizes of the gel bodies were larger than at Au:PPh_3_ ≤ 1:25 ratios even after more than 1 day. Interestingly, the final change in the Prod exponent was smaller for 1:10 (*P * =  1.67) and 1:25 (*P*  =  1.79) than for 1:100 (*P*  =  2.00) (Figure 3c). Decreasing Au:PPh_3_ increased the final Porod exponent, indicating that the wires became more strongly tangled at larger PPh_3_ excess. It is likely that the larger fraction of PPh_3_ in the shell leads to a faster bundle dissolution, increases AuNW polarity, and reduces nanowire‐solvent interactions. The wire‐wire attraction increases, and more tangled, denser gels form.

The above results suggest that a partial replacement of OAm by PPh_3_ is sufficient to induce the dispersion of large into small AuNW bundles and single AuNWs. On the other hand, larger PPh_3_ fractions formed denser gels. To rationalize these results, it is useful to revisit the entropy‐driven AuNW bundling mechanism described by Bettscheider et al.^[^ ^11^, ^12^ ^]^ The authors showed that free ligand molecules bridge sparse OAm ligand shells, cause depletion forces, and induce bundling. This bundling mechanism requires collinear alignment of the AuNWs. Free ligand molecules can partially insert in adjacent AuNW shells and reduce entropy.^[^ ^11^ ^]^ The resulting weak attractive forces accumulate along the contact line and cause bundling in a “zipper‐like” mechanism.^[^ ^15^ ^]^

We propose that the exchange of OAm by PPh_3_ reduces this entropic attraction. Bundles thus disintegrate into smaller bundles and free wires. The steric hindrance of PPh_3_ changes the structure and polarity of the ligand shell, which does not sustain the entropic bridging mechanism anymore. The AuNWs can move apart, and bundles decompose. At the same time, very short‐range enthalpic attraction between PPh_3_‐coated AuNWs increases precisely because the more polar PPh_3_ does not strongly interact with the solvent molecules. The overall effect is a reduced tendency to form regular bundles and an increased tendency to form crosslinked disordered networks. Two AuNWs that touch each other in any orientation are now so attractive that they remain connected, forming a crosslink. Overall, a transition from bundling to gelation takes place. This interpretation explains why the smaller bundles that form during ligand exchange drive out the solvent and cause the macroscopically visible phase separation (Figure 1a). Thinner bundles and single wires can bend with larger curvatures, entangle, and exclude more solvent than the thick bundles that are stiff and strongly interact with the solvent.

A gelation mechanism that is consistent with the above observations proceeds as follows: a partial ligand exchange of OAm on AuNW by PPh_3_ changes surface polarity and surface structure and reduces the entropic bridging of AuNW. This reduces the tendency to form hexagonal bundles and leads to the decomposition of thick bundles into thinner bundles that can bend more easily. At the same time, the progressing ligand exchange reduces the dispersibility of the AuNW, which destabilizes the dispersion, initiates a phase separation of AuNWs from cyclohexane, and increases the local AuNW concentration. The final network is entangled and presumably crosslinked by Van der Waals interactions and π‐π stacking at AuNW crossings that stabilize the observed gel body.

The proposed gelation mechanism is compatible with different relative contributions of nanowire entanglement and crosslinking. The scattering and electron microscopy data presented above do not let us distinguish whether pure entanglement or a combination with physical crosslinks is the underlying reason of the gelation. In the following, we investigate the mechanical properties of the final gels to study the 3D network structure of the gels and to estimate whether pure entanglement or a combination with crosslinks stabilizes it.

**Figure 4:**
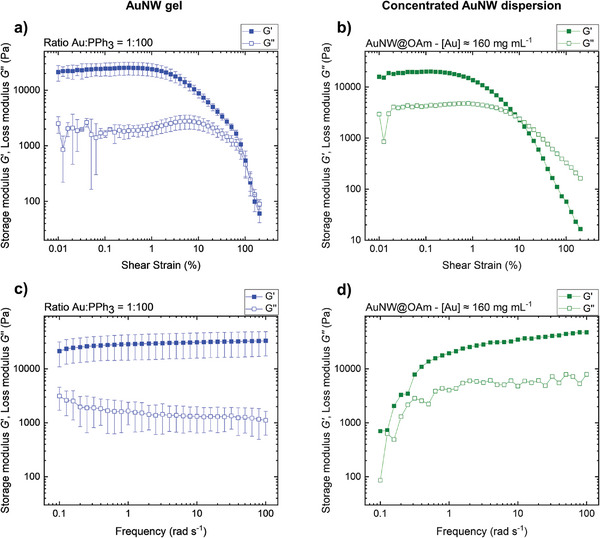
Rheological characterization of chemically formed AuNW gels and highly concentrated AuNW@OAm dispersions at 25 °C. a) Amplitude sweep of an AuNW gel formed at Au:PPh_3_ ratio ≈ 1:100 and b) of a AuNW@OAm dispersion with a gold concentration of ≈160 mg mL^−1^. c) Frequency sweep of a AuNW gel formed at Au:PPh_3_ ratio ≈ 1:100 and d) of a AuNW@OAm dispersion with a gold concentration of ≈160 mg mL^−1^.

The mechanical properties of the gels were investigated by oscillatory rheology. The samples underwent first an amplitude sweep to determine the linear viscoelastic (LVE) region. **Figure** **4**a shows the average and standard deviations of the measured storage and loss moduli (*G*′ and *G*″, respectively) for shear strains between 0.01% and 203% at a frequency of 1 rad s^−1^ for a AuNW gel with a Au:PPh_3_ ratio of 1:100. The graphs for the other ratios can be found in the Supporting Information (Figures S6–S11, Supporting Information).

The storage modulus *G*′ quantifies the deformation energy stored in the sheared sample and reflects its elastic properties. The loss modulus *G*″ represents the energy dissipated by viscous losses.^[^ ^48^ ^]^ The amplitude sweep (Figure 4a) shows that *G*′ > *G*″ in the LVE region, confirming the existence of a gel that behaves as a viscoelastic solid. The gel network uptakes the deformation elastically up to 1.3% ± 0.3% strain (LVE region) before plastic deformations set in.^[^ ^48^ ^]^ The maximum of *G*″ at 6.5% strain is attributed to the dissipation caused by the irreversible deformation of the gel network and resembles the yielding of gels formed from worm‐like micelles, particles, or clays.^[^ ^49^, ^50^, ^51^ ^]^ A maximum in *G*″ and a concurrently decreasing *G*′ indicate the onset of network breakdown;^[^ ^49^, ^50^, ^52^, ^53^ ^]^ the crossover of *G*′ and *G*″ (flow point) at a strain of 120 % marks the breakdown of the network. At higher strains, the material behaved as a viscous fluid. Interestingly, both *G*′ and *G*″ of our AuNW gels decreased at similar rates at higher strains, while most gels exhibit a rapid decrease in *G*′ and a slower decrease in *G*″.^[^ ^52^, ^53^, ^54^ ^]^

The amplitude sweep confirmed the presence of a mechanical network. Such networks can form through interactions between the particles, the ligand shells, or crosslinking, but wires can form gels by simply increasing the volume fractions of the colloid, too.^[^ ^31^ ^]^ We compared the rheology of PPh_3_‐based gels to highly concentrated AuNW@OAm dispersions ([Au] ≈ 160 mg mL^−1^, or 0.8 vol%) in cyclohexane to distinguish these origins of gelation. Figure 4b shows an amplitude sweep of the concentrated AuNW@OAm dispersion with *G*′ > *G*″ in the LVE region that indicates the formation of a gel. Its storage modulus was of a similar order of magnitude as that for PPh_3_‐based gels. The flow point of the concentrated dispersion, however, was at 10% strain, while the PPh_3_‐based gels did not flow until 120% strain (a semi‐logarithmic plot for easier comparison of the flow points is shown in Figure S12, Supporting Information). This shows that pure entanglement is sufficient to create mechanically gel‐like AuNW networks. The gels formed by PPh_3_ had a higher strength, however, despite the lower Au concentrations in the PPh_3_‐based gels of ≈113 mg mL^−1^.

To delve deeper into this difference, we studied the stability of the PPh_3_‐based and AuNW@OAm dispersion with frequency sweeps. Figure 4c shows the averages and standard deviations of *G*′ and *G*″ for frequencies between 0.1 and 100 rad s^−1^ at 0.2% strain of the PPh_3_‐based gels. We found *G*′ > *G*″ in the entire frequency range and only a weak frequency dependence at low frequencies. This is typical for viscoelastic solids such as polymer hydrogels and high‐density crosslinked polymers.^[^ ^48^, ^55^ ^]^ The weak frequency dependence at low frequencies may be due to slight evaporation of solvent during long measurements. Figure 4d shows the frequency sweep for a pure AuNW@OAm dispersion. We also found *G*′ > *G*″ in the entire frequency range but with a strong frequency dependency, where *G*′ dropped by up to 2 orders of magnitude at low frequencies. This is typical for non‐crosslinked polymers.^[^ ^48^ ^]^

The weak frequency dependence of PPh_3_‐based gels (contrary to AuNW@OAm dispersions) indicates crosslinking in these gels. This suggests that gelation is not merely due to entanglement but depends on additional local interactions caused by PPh_3_, most likely a combination of van der Waals attraction and π–π stacking.

**Figure 5:**
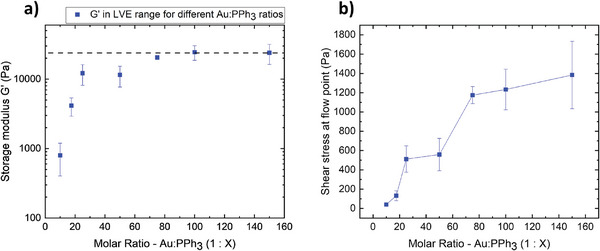
Mechanical strength and stiffness of the PPh_3_‐based gels as a function of Au:PPh_3_. The plots show a) storage modulus (*G*′) and b) shear stress at the flow point of different AuNW gels dependent on the Au:PPh_3_ ratio.

We studied the effect of Au:PPh_3_ on the mechanical properties of the PPh_3_‐based gels. Gels formed at Au:PPh_3_ ratios of 1:10, 1:17.5, 1:25, 1:50, 1:100 and 1:150 were characterized 18–20 h after gelation using amplitude sweeps. All gels showed similar LVE regions with a viscoelastic solid behavior characterized by *G′ > G″* (Figure S6–S11, Supporting Information). The average *G'* values in the LVE region (**Figure** **5**a) increased by up to one order of magnitude when decreasing Au:PPh_3_ from 1:10 to 1:25. They further increased and reached a plateau of *G*′  =  25 kPa at Au:PPh_3_ ≈ 1:75. The shear stress at the flow point, where *G*′ and *G*″ cross and the network collapses, also increased with decreasing Au:PPh_3_ ratio (Figure 5b). At high ratios (1:10), the shear stress at the flow point was low and increased sharply by more than one order of magnitude up to a ratio of 1:75. At lower ratios than 1:75, the shear stress at the flow point changed only slightly, similar to the change of *G*′. This shows an increase of stiffness and resilience of the gels with decreasing Au:PPh_3_ up to a ratio of 1:75 and is consistent with a PPh_3_‐dependent crosslinking that saturates at a maximum density, either because all ligands are replaced or because the overall network geometry does not allow for additional crosslinks.

In summary, our rheological analysis confirmed the PPh_3_‐induced gelation mechanism proposed above and suggested that crosslinking stabilizes the network structure. It was possible to tune the properties of the AuNW gels through the degree of ligand exchange, probably because PPh_3_ density affects the gel density and the strength and number of crosslinks.

## 3 Conclusion

We synthesized organic‐inorganic hybrid gels of ultrathin gold nanowires with diameters below 2 nm. Gelation was chemically induced by ligand exchange of OAm against PPh_3_. The gelation and the mechanical properties were dependent on the Au:PPh_3_ ratio. We propose a PPh_3_‐induced multi‐step gelation mechanism that is consistent with all observations from SAXS, TEM, and rheological analysis. The replacement from OAm to PPh_3_ on the nanowire surface increased ligand‐ligand and reduced ligand‐solvent interactions, resulting in a destabilization of the dispersion and phase separation. Thick hexagonal AuNW bundles dispersed during gelation. Thinner bundles and individual nanowires are entangled and crosslinked. Rheological analyses show the crosslinking and that the PPh_3_‐based gels are more resilient than purely entanglement‐based gels formed from high concentrated AuNW@OAm dispersions. It is likely that Van der Waals interactions and π–π stacking of the PPh_3_ ligands led to the crosslinking. We were able to tune the mechanical properties of PPh_3_‐based gels with the Au:PPh_3_ ratio. Lower ratios led to stiffer gels with higher strength.

## 4 Experimental Section

### Chemicals

*N*‐hexane (≥99 %) and cyclohexane (≥99.5 %) were purchased from Carl Roth (Karlsruhe, Germany). Triphenylphosphine (PPh_3_, 95 %) and gold(III)chloride trihydrate (HAuCl_4_·3H_2_O, ≥99.9 %) were obtained from Thermo Fisher Scientific (Waltham, MA, USA) and triisopropylsilane (TiPS, 98 %) was purchased from Abcr GmbH (Karlsruhe, Germany). Absolute ethanol (99.95 %) was used from VWR (Radnor, PA, USA) and OAm (80%–90%) from Acros Organics (Geel, Belgium). All chemicals were used without further purification.

### AuNW Synthesis

AuNWs were synthesized following a protocol published by Feng et al., Loubat et al., and Nouh et al., with minor modifications.^[^ ^10^, ^13^, ^14^ ^]^ A 25 mL glass vial was filled with solid HAuCl_4_·3H_2_O (40 mg, 0.102 mmol), and *n*‐hexane (6.6 mL) was added quickly. Then OAm (1.36 mL, 4.134 mmol) was added, and the mixture was shaken under an argon atmosphere with a vortex until the solid had dissolved. After this, TiPS (2.04 mL, 9.926 mmol) was added and the vessel was flushed with argon, sealed, and agitated vigorously for a few minutes. The reaction mixture was then stored without stirring at 25 °C for 24 h. During this, the color of the mixture changed from deep orange to a slight yellow, and finally, the brownish color typical of dispersions of AuNWs.

For purification, the nanowires were precipitated by adding twice the volume of EtOH to the dispersion and centrifugated at 40 rcf for 3 min. The supernatant was removed with a pipette and the residue was resuspended in 10 mL cyclohexane. This protocol was repeated at 172 rcf for 3 min and the residue was again redispersed in 10 mL cyclohexane. After both purification steps a AuNW dispersion [Au] ≈ 2 mg mL^−1^ was obtained. The concentration was calculated according to the amount of used HAuCl_4_·3H_2_O and verified using thermogravimetry.

### Gel Formation

A 4 mL cylindrical glass vial was filled with 1 mL of the AuNW dispersion (2 mg mL^−1^) in cyclohexane and 1 mL of cyclohexane solutions containing different amounts of PPh_3_. The mixture was vortexed for 30 s and left standing overnight in the sealed vial. A stable body of gel that was slightly smaller than the vessel formed and floated at the bottom of cyclohexane. Molar Au:PPh_3_ ratios of 1:100 (266 mg mL^−1^ PPh_3_ in cyclohexane) was used for the TEM studies; 1:10 (27 mg mL^−1^ PPh_3_ in cyclohexane), 1:25 (67 mg mL^−1^ PPh_3_ in cyclohexane) and 1:100 (266 mg mL^−1^ PPh_3_ in cyclohexane) for SAXS experiments; and 1:10 (27 mg mL^−1^ PPh_3_ in cyclohexane), 1:25 (67 mg mL^−1^ PPh_3_ in cyclohexane), 1:50 (133 mg mL^−1^ PPh_3_ in cyclohexane), 1:75 (199 mg mL^−1^ PPh_3_ in cyclohexane), 1:100 (266 mg mL^−1^ PPh_3_ in cyclohexane), 1:150 (399 mg mL^−1^ PPh_3_ in cyclohexane) for the rheological studies. The concentration of AuNWs in the precursor solution was kept constant for all gels.

### Freeze‐Drying of the AuNW Gel

After gelation excess solvent was removed with a glass pipette and replaced with the same amount of pure cyclohexane. This solvent exchange was repeated 2 times. The gel and solvent were then flash‐frozen in a 50 mL round bottom flask using liquid nitrogen. This flask was connected to a P5‐85‐KB freeze dryer (Piatkowski Forschungsgeräte, Petershausen, Germany) and the solvent was removed over 24 h at 0.23 mbar.

### In Situ Small Angle X‐Ray Scattering (SAXS)

The gelation process of the AuNWs was tracked in situ using SAXS using a Xeuss 2.0 setup from Xenocs (SAS, Grenoble, France). The incident X‐ray beam from a Copper K_α_ source (wavelength *λ*  =  0.154 nm) was collimated and focused on the sample with a spot size of 0.25 mm^2^. 2D scattering patterns were recorded with a PILATUS3 R 1 m detector (Dectris, Baden, Switzerland) at a sample‐detector distance of *l* ≈ 1.02 m and calibrated using a silver behenate standard. The AuNW dispersion and PPh_3_ solution were mixed as described above, immediately transferred to a borosilicate capillary with a diameter of 1.5 mm, and placed in the SAXS setup. Consecutive SAXS patterns were obtained with acquisition times of 15 min. The software XSACT 2.7.1 (Xenocs SAS, Grenoble, France) was used to azimuthally average the 2D scattering patterns to obtain the scattered intensity *I* in dependence on momentum transfer, *q*, given by Equation (2):

(2) $$q=\frac{4\pi }{\lambda }\sin\left(\theta /2\right)$$

with *θ* being the scattering angle. Background scattering was subtracted using a reference measurement of pure solvent. The overall model that in fitting the radially averaged scattering is based on reports by Förster et al., Loubat et al., and Sundblom et al. was used and is detailed in the Supporting Information.^[^ ^13^, ^56^, ^57^ ^]^

### Rheological Measurements

Rheological properties of the AuNW gels were measured using a MCR 302e rheometer (Anton Paar, Austria) with a parallel plate configuration. The excess solvent was removed from the vessel containing the gel and the solid gel piece was placed on the rheometer. The solvent‐soaked gel was confined between two steel plates with 15 mm diameter of the top plate and a gap of 100 µm. Evaporation, drying of the organogel, and precipitation of PPh_3_ was prevented through a saturated solvent atmosphere. All measurements were performed in oscillating deformation mode at 25 °C after a resting time of 30 s to eliminate the effects of loading history. Stresses were measured as a function of shear strain at a constant frequency of 1 rad s^−1^ (amplitude sweep) and as a function of frequency at a constant shear strain of 0.2 % (frequency sweep).

### Transmission Electron Microscopy

Samples of 30 µL were removed from the forming gel, drop‐casted onto a carbon‐coated copper grid (Plano, Wetzlar, Germany) using a micropipette, and dried in air for several hours. Samples were taken at 0 , 15 , 30 , 60 , 180 , and 240 min after mixing the AuNW dispersion and the PPh_3_ solution of 266 mg mL^−1^ in cyclohexane. The samples were observed at an acceleration voltage of 200 kV in a JEM 2100 LaB_6_ microscope (JEOL, Akishima, Japan). TEM images were captured using an Orius SC1000 CCD camera (Gatan, Pleasanton, CA, USA) at an exposure time of 2 s.

### Scanning Electron Microscopy

SEM were recorded with a Quanta 400 ESEM (FEI Technologies Inc., Oregon, United States) in a low or in a high vacuum mode with an acceleration voltage of 15 and 20 kV. All contrast was from secondary electron detection. Images in low vacuum were recorded with a LFD detector (FEI Technologies Inc., Oregon, United States), in high vacuum with an ETD detector (FEI Technologies Inc., Oregon, United States).

## Conflict of Interest

The authors declare no conflict of interest.

## Supporting information



Supporting Information

## Data Availability

The data that support the findings of this study are available from the corresponding author upon reasonable request.
