# Supplementary material for: Hybrid Ultrathin Gold Nanowire Gels: Formation and Mechanical Properties
Source: Small. 2025 Mar 3;21(14):2411506. doi: 10.1002/smll.202411506 (PMC11983238; doi:10.1002/smll.202411506)
Supplement: Supplementary file 1 — Supporting Information [file SMLL-21-2411506-s001.docx]

Supporting Information

Hybrid ultrathin nanowire gels: formation and mechanical properties

Yannic Curto, Srishti Arora, Bart-Jan Niebuur, Lola González-García, and Tobias Kraus*

Yannic Curto, Srishti Arora, Bart-Jan Niebuur

INM –Leibniz Institute for New Materials, Campus D2.2, Saarbrücken 66123, Germany

Lola González-García

INM –Leibniz Institute for New Materials, Campus D2.2, Saarbrücken 66123, Germany

Department of Materials Science and Engineering, Saarland University, Campus D2.2, 66123, Saarbrücken, Germany

Tobias Kraus

INM –Leibniz Institute for New Materials, Campus D2.2, Saarbrücken 66123, Germany

Colloid and Interface Chemistry, Saarland University, Campus D2.2, Saarbrücken 66123, Germany
E-mail: tobias.kraus@leibniz-inm.de

**Fitting model of the SAXS curves.**

Following an approach described previously ^[1 – 3]^, all SAXS patterns of nanowire dispersions were modelled using Equation S1

$I\left( q \right)=I_{\mathrm{NW}}\left( q \right)S_{\mathrm{hex}}\left( q \right)+I_{\mathrm{bkg}}$ (S1)

with *I*_NW_ (*q*) the form factor of nanowires, *S*_hex_ (*q*) a structure factor accounting for their arrangement in 2D-hexagonal bundles and *I*_bkg_ a constant background.

*I*_NW_ (*q*) (Equation S2) is approximated by the product of the form factor of infinitely thin rods, *P*_rod_ (*q*), and the circular cross-section form factor, *P*_CS_ (*q*), as

$I_{NW}\left( q \right)=CP_{\mathrm{rod}}\left( q \right)P_{\mathrm{CS}}\left( q \right)$ (S2)

with *C* a constant accounting for the arbitrary intensity scaling of the measurements. Here, *P*_rod_ (*q*) is given Equation S3.

$P_{\mathrm{rod}}\left( q \right)= L^{2}\left( \frac{2\cdot\mathrm{Si}\left( qL \right)}{qL}-4\frac{\sin^{2}\left( \frac{qL}{2} \right)}{\left( qL \right)^{2}} \right)$ (S3)

with *L* the length of the nanowires, and Si (*x*) the sine integral, given Equation S4.

$\mathrm{Si}\left( x \right)=\int_{0}^{x} \frac{\sin x'}{x'}dx'$ (S4)

To account for polydispersity in the nanowire radius, *P*_CS_ (*q*) (Equation S5) is determined by convoluting the circular cross-section scattering amplitude, *F*_CS_ (*q*,*R*) (Equation S6), with a Schulz-Zimm distribution function, *D* (*R*), as

$P_{\mathrm{CS}}\left( q \right)=\int_{0}^{\infty} D\left( R \right)F_{CS}^{2}\left( q,R \right)dR$ (S5)

with

$F_{\mathrm{CS}}\left( q,R \right)=\pi R^{2}\frac{2J_{1}\left( qR \right)}{qR}$ (S6)

Here, *R* denotes the radius of the nanowires. *D* (*R*) is given Equation 7.

$D\left( R \right)=\left( z+1 \right)^{z+1}X^{z}\frac{\exp\left( -\left( z+1 \right)X \right)}{R_{\mathrm{avg}}\Gamma\left( z+1 \right)}$ (S7)

with $X=R/R_{\mathrm{avg}}$, $R_{\mathrm{avg}}$ the average nanowire core radius, $z=\frac{1}{p^{2}}-1$ with $p=\frac{\sigma_{R}}{R_{\mathrm{avg}}}$, and $\sigma_{R}^{2}$ the variance of the nanowire core radius distribution.

*S*_hex_ (*q*) describes a 2D hexagonal superstructure of nanowires, and is given by Equation S8

$S_{\mathrm{hex}}\left( q \right)=1-\beta\left( q \right)G\left( q \right)+\beta\left( q \right)G\left( q \right)Z_{0}\left( q \right)$ (S8)

The lattice factor, *Z*_0_ (*q*), describes scattering by the lattice, which, for a 2D-hexagonal lattice, is given by Equation S9

$Z_{0}\left( q \right)=\frac{2}{\sqrt{3}a^{2}q}\sum_{hk} m_{hk}L_{hk}\left( q,q_{hk} \right)$ (S9)

where *a* is the unit cell dimension, which, for a 2D-hexagonal lattice, equals the closest distance between next neighboring particles. *m*_hk_ is the multiplicity of each peak determined by Miller indices *h* and *k*, which equals 6 (h≠0 and k=0, h=k≠0) or 12 (h≠k≠0). The peak shape functions *L*_hk_ (*q*, *q*_hk_) are centered at the peak positions $q_{hk}=\frac{4\pi}{\sqrt{3}a}\sqrt{h^{2}+hk+k^{2}}$ and are approximated by a Lorentz distribution, given by Equation S10

$L_{hk}\left( q,q_{hk} \right)= \frac{\left( \frac{\delta}{2\pi} \right)}{\left( q-q_{hk} \right)^{2}+\left( \frac{\delta}{2} \right)^{2}}$ (S10)

The Debye-Scherrer equation relates the peak width, *δ*, to the domain size or the average bundle diameter, *D*_bundle_ (Equation S11), as

$D_{\mathrm{bundle}}=\frac{2\pi}{\delta}$ (S11)

The factor *β (q)* accounts for the influence of nanowire polydispersity on their arrangement in bundles, and is given by Equation S12

$\beta\left( q \right)=\frac{\left\langle F_{\mathrm{CS}}\left( q \right) \right\rangle^{2}}{\left\langle F_{\mathrm{CS}}^{2}\left( q \right) \right\rangle}$ (S12)

With

$\left\langle F_{\mathrm{CS}}\left( q \right) \right\rangle=\frac{\int_{0}^{\infty} D\left( R \right)F_{\mathrm{CS}}\left( q,R \right)dR}{\int_{0}^{\infty} D\left( R \right)dR}$ (S13)

And

$\left\langle F_{\mathrm{CS}}^{2}\left( q \right) \right\rangle=\frac{\int_{0}^{\infty} D\left( R \right)F_{\mathrm{CS}}^{2}\left( q,R \right)dR}{\int_{0}^{\infty} D\left( R \right)dR}$ (S14)

Analogous to the Debye-Waller factor in case of attenuation of the scattering amplitude due to atomic vibrations in crystal lattices, positional disorder of the nanowire in bundles is accounted by the disorder parameter, *G (q)*, given by Equation 15

$G\left( q \right)=\exp\left( -q^{2}a^{2}\sigma_{a}^{2} \right)$ (S15)

where *σ_a_* denotes the standard deviation of the mean distance between nanowires.

**SAXS and microscopy of oleylamine capped AuNWs:**

SAXS curve (Figure S1a) and TEM image (Figure S1b) of AuNW@OAm were used to analyze the shape and arrangement of the nanowires in the dispersion. The fit (with the model above) of the scattering curve shows an average AuNW diameter of 1.9 ± 0.3 nm and an average center-to-center distance of the nanowires in bundles *d*_c-c_ of 5.9 nm. Figure S1c shows the size distribution of the AuNW diameter with an average diameter of 1.6 ± 0.1 nm, which was determined out of TEM images vis Image J.


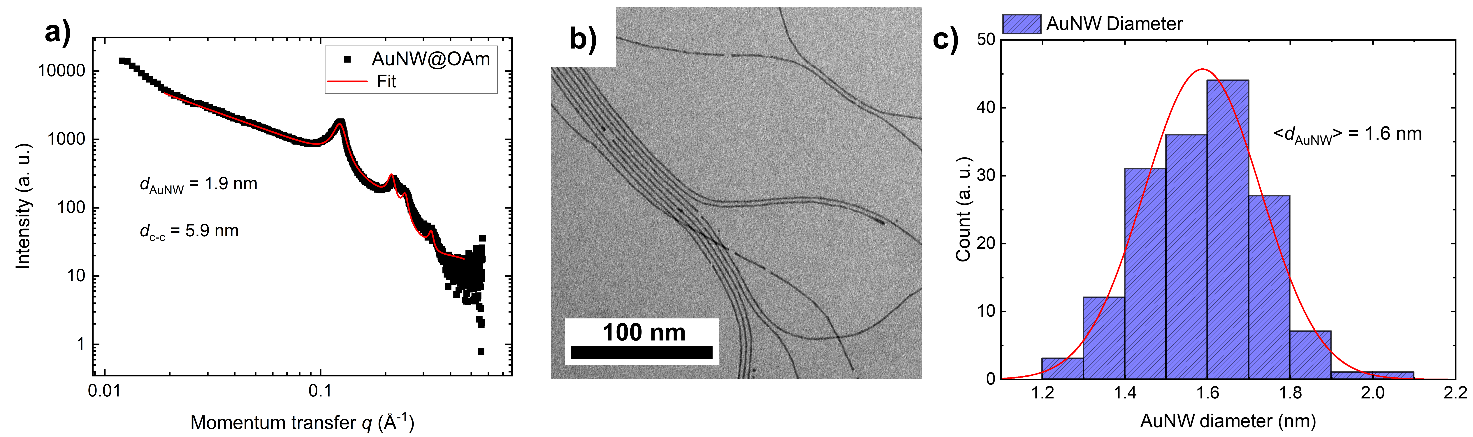


**Figure S1.** SAXS curve and TEM imaging of the initial AuNW@OAm dispersion used to determine the wire diameter. a) SAXS curve (0.01 – 0.56 Å^-1^) of the initial AuNW@OAm dispersion and the associated fitting curve to determine the average AuNW diameter and center-to-center distance of the AuNWs in bundles. b) TEM image of AuNW with OAm as a stabilizing ligand. c) Size distribution of the AuNW diameter determined via Image J from TEM images.

**Light and electron microscopy of AuNW gel dispersed in MeOH:**

Light microscopy image (Figure S2a) and TEM image (Figure S2b) of redispersed AuNW gel in methanol showing that the dispersed gel contains small agglomerates of entangled nanowires instead of completely non agglomerated nanowires.


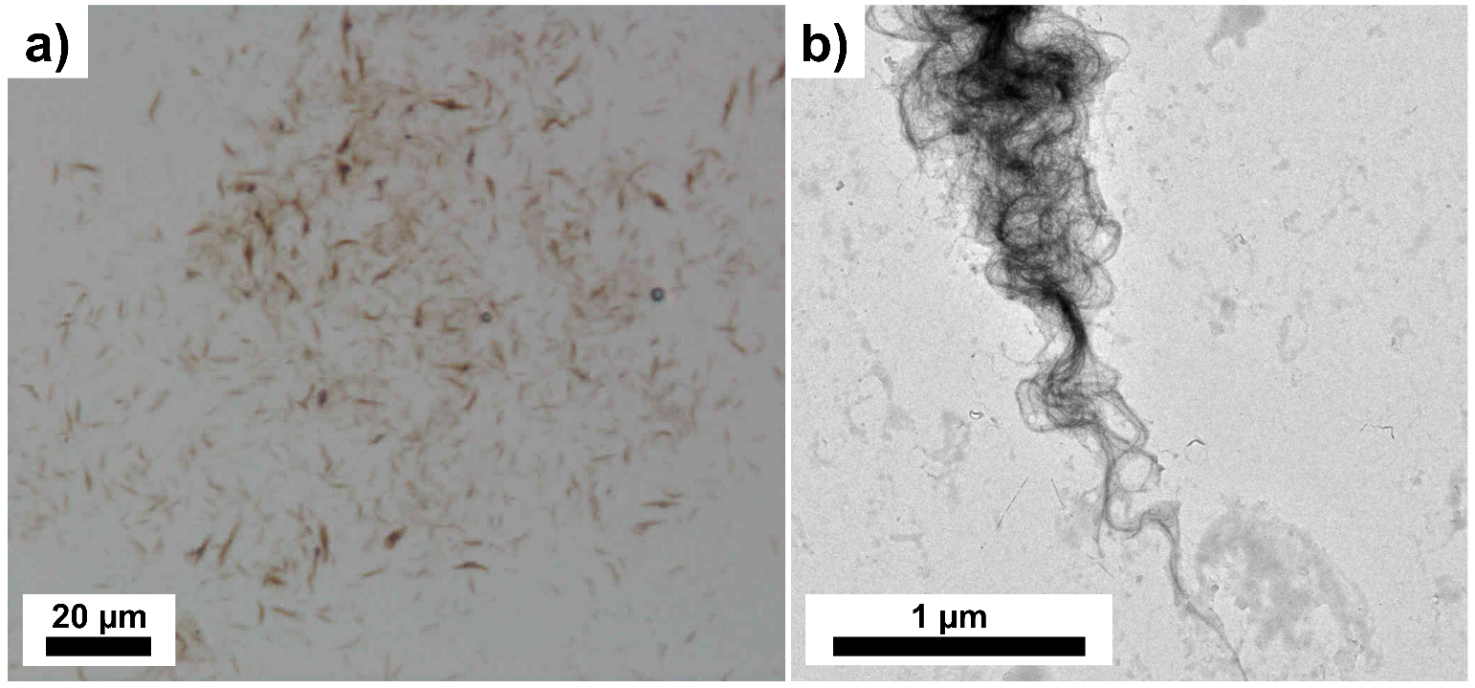


**Figure S2.** Imaging of the AuNW gel suspended in methanol on different length scales. a) Light microscopy image of the AuNW gel (Au:PPh_3_ ratio of 1:100) suspended in methanol. b) TEM image of the AuNW gel (Au:PPh_3_ ratio of 1:100) suspended in methanol.

**TEM image of the AuNW gel after a gelation time of 1 day:**

TEM images were taken from samples obtained after a gelation time of 1 day. They were qualitatively similar to those taken after 2-4 h. No significant changes of the nanowires and their bundles were observed; small bundles of a few nanowires and free nanowires are visible in Figure S3.


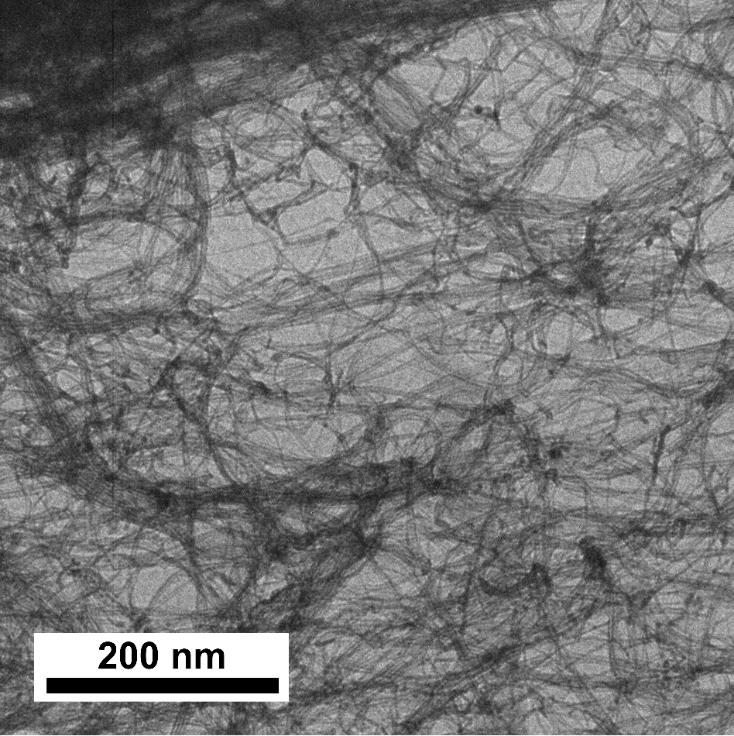


**Figure S3.** TEM image of a AuNW gel (Au:PPh_3_ = 1:100) after a gelation time of 1 day.

**In situ SAXS of the gelation:**

In situ SAXS studies of the gelation of AuNW (Au:PPh_3_ ratio of 1:00) in a range of *q*-values between 0.035 and 1 Å^-1^ (Figure S4) yielded secondary, tertiary and quaternary structure factor peaks of the AuNW bundles. Their presence indicates the hexagonal superstructure of the nanowires in the bundles. We found that all peaks of the structure factor decreased with time during gelation as shown for the primary structure factor peak in Figure 3a. The implications for the gelation mechanism are discussed in the main text.

**Figure S4.** SAXS patterns of the AuNW gelation process with Au:PPh_3_ ratio of 1:100 depending on time after the initiation of the gelation process. The SAXS curves measured at higher momentum transfer of 0.035 – 1 Å^-1^ to also show the secondary, tertiary and quaternary structure factor peaks during the gelation.

The scattering curves of the in situ SAXS measurements for Au:PPh_3_ ratios of 1:10 (Figure S5a) and 1:25 (Figure S5b) showed similar curve profiles as for 1:100 (Figure 3a) but the decrease of the structure factor peak was slower, especially for a Au:PPh_3_ ratio of 1:10 (Figure S5a), where the structure factor peak did not vanish completely after 915 min.


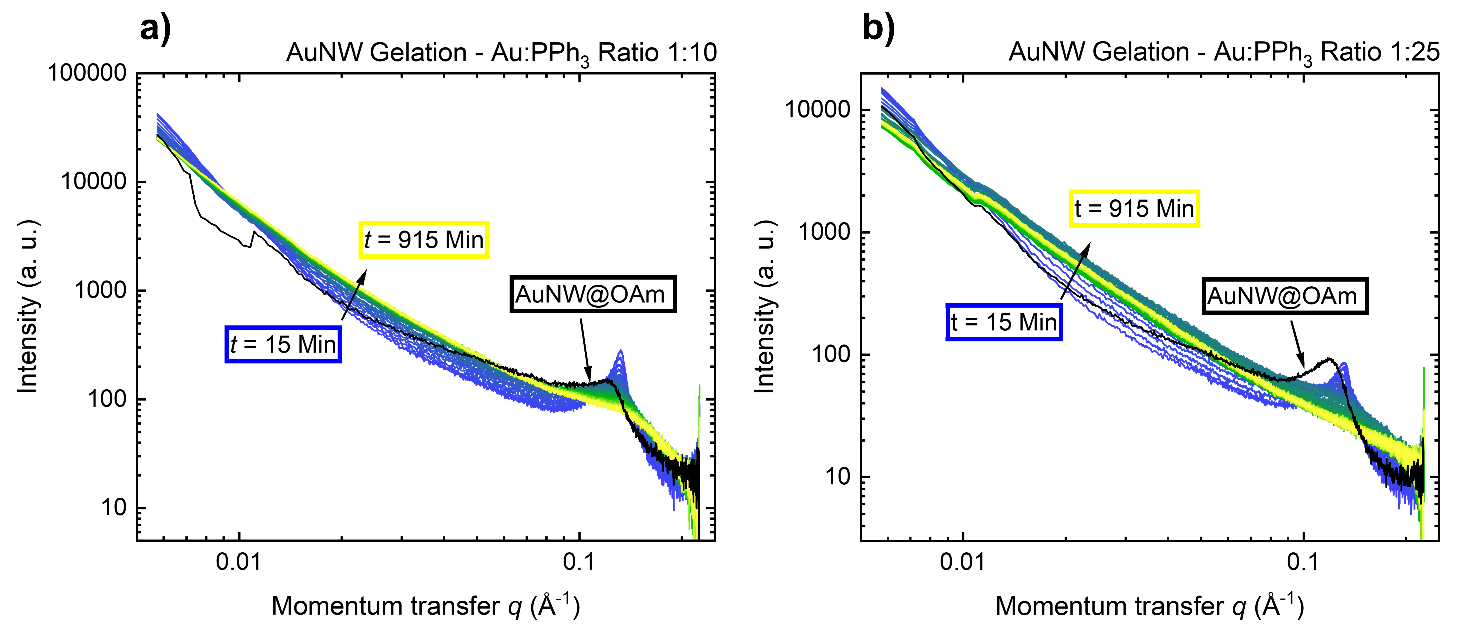


**Figure S5.** SAXS patterns obtained during the AuNW gelation process with Au:PPh_3_ ratios of a) 1:10 and b) 1:25 at different times after the start of the gelation process as indicated in the graphs with a color gradient from blue to yellow.

**Rheology of AuNW gels:**

The mean amplitude sweeps of AuNW gels with Au:PPh_3_ ratios of 1:10, 1:17.5, 1:25, 1:50, 1:75 and 1:150 show similar curve profiles as for Au:PPh_3_ ratio of 1:100 but with different storage moduli G’ dependent on the Au:PPh_3_ ratio. This is described in detail in the main text.

**Figure S6.** Amplitude sweep of AuNW gel with a Au:PPh_3_ ratio of 1:10. Storage and loss modulus dependent on shear strain.

**Figure S7.** Amplitude sweep of AuNW gel with a Au:PPh_3_ ratio of 1:17.5. Storage and loss modulus dependent on shear strain.

**Figure S8.** Amplitude sweep of AuNW gel with a Au:PPh_3_ ratio of 1:25. Storage and loss modulus dependent on shear strain.

**Figure S9.** Amplitude sweep of AuNW gel with a Au:PPh_3_ ratio of 1:50. Storage and loss modulus dependent on shear strain.

**Figure S10.** Amplitude sweep of AuNW gel with a Au:PPh_3_ ratio of 1:75. Storage and loss modulus dependent on shear strain.

**Figure S11.** Amplitude sweep of AuNW gel with a Au:PPh_3_ ratio of 1:150. Storage and loss modulus dependent on shear strain.

**Flow points of chemically gelled AuNW compared to highly concentrated dispersions:**

Figure S12 shows the amplitude sweeps of a PPh_3_-based AuNW gel (Au:PPh_3_ ≈ 1:100) and a highly concentrated AuNW@OAm dispersion ([Au] ≈ 160 mg mL^-1^) with a linear x-axis that makes it easier to compare the flow points of the two samples. It is apparent that the highly concentrated dispersion flows at approximately 10 % strain, whereas the flow point of the PPh_3_-based gel is significantly higher at around 120 %. This difference in mechanical properties is discussed in the main text.

**
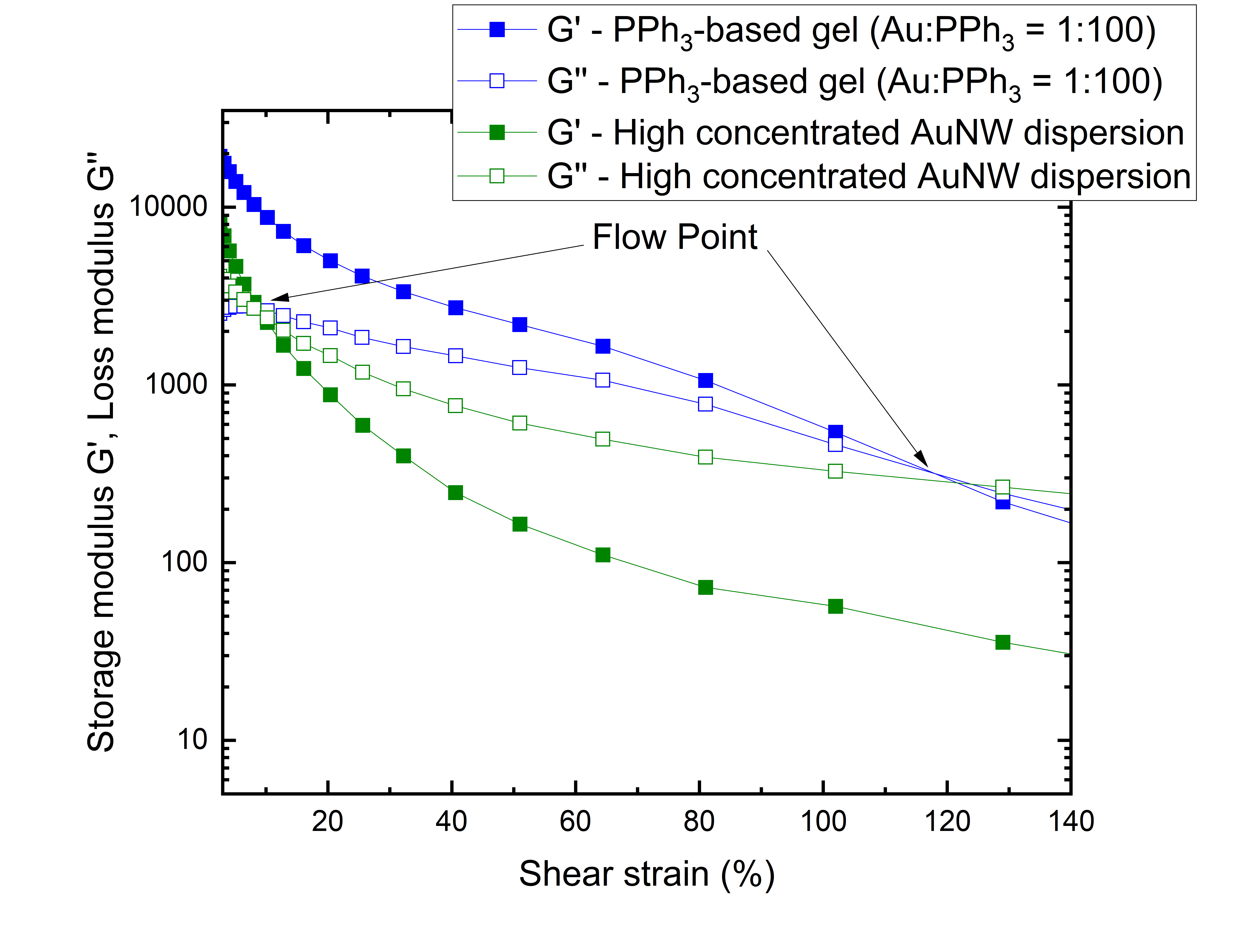
**

**Figure S12.** Amplitude sweeps of a PPh_3_-based AuNW gel at Au:PPh_3_ of 1:100 and a highly concentrated AuNW@OAm dispersion ([Au] ≈ 160 mg mL^-1^). The chemically induced AuNW gel started flowing at significantly larger shear strains.

References

[1] A. Loubat, M. Impéror-Clerc, B. Pansu, F. Meneau, B. Raquet, G. Viau, L. M. Lacroix, *Langmuir* **2014**, *30*, 4005.

[2] S. Förster, A. Timmann, M. Konrad, C. Schellbach, A. Meyer, S. S. Funari, P. Mulvaney, R. Knott, *J. Phys. Chem. B* **2005**, *109*, 1347.

[3] A. Sundblom, C. L. P. Oliveira, A. E. C. Palmqvist, J. S. Pedersen, *J. Phys. Chem. C* **2009**, *113*, 7706.
